# Elite Triathlete Profiles in Draft-Legal Triathlons as a Basis for Talent Identification

**DOI:** 10.3390/ijerph19020881

**Published:** 2022-01-13

**Authors:** Alba Cuba-Dorado, Tania Álvarez-Yates, Oscar García-García

**Affiliations:** Laboratory of Sport Performance, Physical Condition and Wellness, Faculty of Education and Sport Sciences, University of Vigo, Campus A Xunqueira s/n, 36005 Pontevedra, Spain; tanalvarez@uvigo.es (T.Á.-Y.); oscargarcia@uvigo.es (O.G.-G.)

**Keywords:** triathlon, sport performance, anthropometry, physiology, biomechanics

## Abstract

Draft-legal triathlons are the main short-distance races worldwide and are those on which talent-identification programs are usually focused. Performance in these races depends on multiple factors; however, many investigations do not focus on elite triathletes. Therefore, the aim of this narrative review was to carry out a systematic literature search to define the elite female and male triathlete profiles and their competition demands in draft-legal triathlons. This will allow us to summarize the main determinant factors of high-level triathletes as a basis for talent detection. A comprehensive review of Web of Science and Scopus was performed using the search strategy: Triathl* and (performance or competition or profile) and (elite or professional or “high performance” or “high level” or talent). A total of 1325 research documents were obtained, and after screening following the criteria, only 83 articles were selected. After data synthesis, elite triathlete aspects such as age, physiological, anthropometric, and psychosocial profile or competition demands were studied in the scientific literature. Thus, it is essential that when implementing talent identification programs, these factors must be considered. However, constant updating is needed due the continuous regulatory changes and the need of triathletes to adapt to these new competition demands.

## 1. Introduction

Triathlon is a sport that combines three disciplines: swimming, cycling, and running. These are carried out consecutively and in this order. Between each discipline, there is a transition where each triathlete changes equipment to face the next discipline. Therefore, triathletes must complete two transitions, the first from swimming to cycling (T1), and the second from cycling to running (T2).

There are different official competitions depending on race distances (see Table 1), all regulated by *World Triathlon*, the highest organization worldwide that regulates official triathlon competitions.

In triathlon races, it is important to highlight the “drafting” concept, used in sport physiology and biomechanics to describe taking a tactical position behind other triathletes to obtain a benefit. Since the decision in 1994 to include Triathlon in Sydney 2000 Olympic Games, drafting is allowed in the cycling segment, initiating the “drafting period” in official short distances. However, in longer triathlons, such as middle and long distances, cycling drafting is not allowed. This is one of the clearest differentiating elements of triathlon distances.

Given the multidisciplinary nature of triathlon, “triathlon is more than the sum of its parts” [1]. The interaction between its components (swimming, T1, cycling, T2 and running) must be considered for the overall performance analysis, which will depend on the somatotype, physiological capacity, mastery of technique, or running strategy of the triathlete [2].

Scientific analysis of triathlon performance initially focused on long distance triathlon until the late 1990s, when it entered in the Olympic program. This led to an increase in short distance research, especially conducted by a group of French researchers, considered the pioneers [3]. Since then, many researchers have tried to point out triathletes’ characteristics by focusing on their physiological and anthropometric aspects or race strategies. However, many of these investigations did not focus on elite triathletes, including, in many cases, amateurs or high-performance aspirants. We consider it important to distinguish between performance levels and to know what factors have a direct impact on high performance. In the same way, it seems necessary to differentiate between female and male triathletes’ characteristics. Therefore, it seems interesting to carry out a systematic review that focuses only on elite triathletes and differentiates information based on gender, both for a good athletic development and as a reference for their special characteristics.

Having reference characteristics of female and male triathletes who achieve the highest performance will undoubtedly be important for developing optimal training programs and for being able to recognize talented triathletes that can reach the highest performance at an early age—in other words, to identify triathletes with potential to become elite athletes [4]. The knowledge of these characteristics will be the first step to design fair talent development programs that combine many aspects and components to achieve an optimal development of the triathlete. There is still much ignorance about talent predictors for elite sport, and even more about talent predictors in women [5]. Therefore, it seems necessary to provide valuable information with scientific rigor for those in charge of talent identification programs (i.e., federations, sport institutions, coaches, national selectors, etc.) to develop them in the most optimal way possible.

Therefore, the aim of this narrative systematic review was to carry out a search of the triathlon scientific literature focused on clearly defining the elite triathlete profile and the competition demands in draft-legal triathlons. This will allow us to summarize the main determinant factors of high-level triathletes as a basis for talent detection.

## 2. Materials and Methods

### 2.1. Literature Search

A comprehensive review of the scientific literature was carried out between September and October 2021 through Scopus and Web of Sciences databases using the search strategy: Triathl* and (performance or competition or profile) in topics and (elite or professional or “high performance” or “high level” or talent) in all fields. These terms were used to collect all published research documents on triathlons, but we limited ourselves only to the target population (elite female and male triathletes) to discern from the existing evidence on amateur-, regional- or national-level athletes.

All research documents either in English (75), Spanish (7) or Portuguese (1) that provided differentiated data from elite triathletes, such as conference presentations or posters (3), short communications (1), original investigations (69), reviews (8), invited commentaries (1) and editorial comments (1), were included in this review.

Investigations regarding triathlon performance-limiting factors (i.e., injuries), environmental factors, and modifiable aspects (i.e., nutritional strategies, supplementation and doping, training methods, recovery, aerodynamics, and material technology) were considered exclusive criteria. However, if they provided valuable data to characterize the elite female or male triathlete profile, the exclusion was reconsidered. Meanwhile, studies that (a) did not specify triathletes’ gender (whether they were females or males), (b) did not report triathletes’ age (in non-competition conditions), and (c) did not provide differentiated data between triathletes and other study participants, were also excluded. Any discrepancies between the two researchers were resolved by reaching consensus. If consensus was not possible, a third researcher resolved the discrepancy (O.G.).

### 2.2. Selection of Research Documents

**Figure 1:**
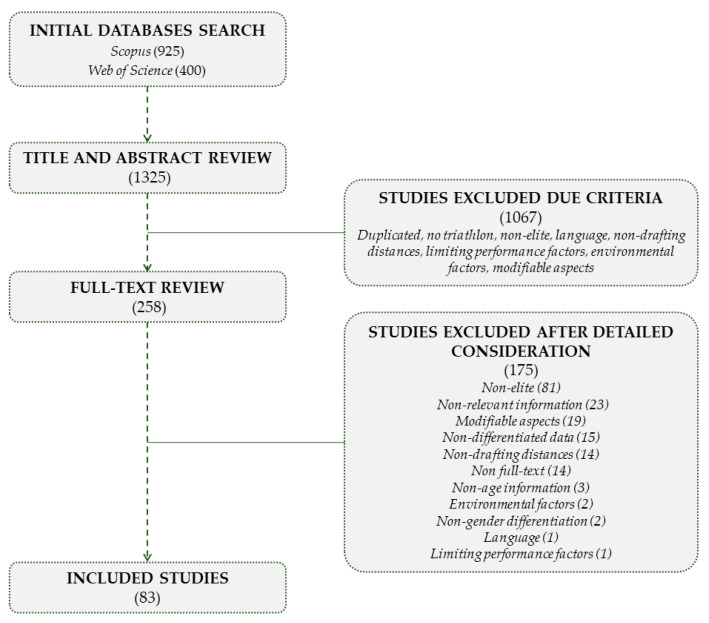
Flow chart for study selection.

The results from the literature search are synthesized in Figure 1. Through the search strategy described above, 1325 research documents were obtained, analyzing and selecting only those that met the inclusions criteria: (a) included a sample with elite or high-level triathletes, (b) outlined value data to characterize female and male elite triathletes, (c) reported triathletes’ behavior in official top-level races (i.e., Triathlon World Championships, Triathlon World Cups, Final World Triathlon Series events, Triathlon Continental Championships), (d) provided knowledge about high triathlon performance, (e) pointed out information regarding high performance prediction, and (f) contributed to talent identification processes, highlighting relevant aspects to detect or develop young triathletes.

After title and abstract screening, 258 documents remained for further full-text analysis. Finally, a total of 83 documents qualified for inclusion after excluding 175 documents due detailed consideration after full-text review (did not meet the inclusion criteria, did not report triathlete gender or age, no full text available, non-drafting triathlon, or did not directly provide relevant information to characterize elite triathletes’ profiles) (see Figure 1). The selected studies were mainly published between the 2000s (35) and the 2010s (33), although the first publications in this matter began to appear in the 1990s (6). Nine research documents from the 2020s were included following the criteria.

## 3. Results and Discussion

Numerous studies addressed the different factors that have a direct impact on high-level triathlon performance in sprint and standard triathlon distances.

The elite female and male triathlete profile that characterizes the effort and demands of sprint and standard distance triathlons requires different physiological, anthropometric, biomechanical, and tactical factors.

### 3.1. Elite Triathle Competitive Age

Various studies analyzed the age of triathletes participating in top-level competitions. Villaroel et al. [6] concluded that the optimal age range to obtain a male top 10 rank at the WTS level is between 26 and 32 years old, suggesting that experience is a very important factor for Olympic Games performance, since they observed a higher age average in these races. In this line, Malcata et al. [7] and Werneck et al. [8] indicated that the age of maximum performance for males and females was 28 ± 2 and 27 ± 4 years, respectively, while Knechtle et al. [9] reported ages of 27.1 ± 4.9 for males and 26.6 ± 4.4 years for females. Based on these studies, we can conclude that the age of maximum performance in triathlon is around 27 years in both females and males.

Werneck et al. [8] delved deeper into the study of age in triathlons, observing that most Olympic triathletes, especially male, were born in the first quarter of the year (32% in the first quarter, 30% in the second, 21% in the third, and 17% in the fourth). Similarly, these authors revealed that 80% of male triathlete medalists in Olympic Games were born within the first quarter of the year, while females who were born in the first quarter obtained the same number of medals as those born in the second quarter.

Based on the above, it seems that approximately 30 is the age at which performance is optimized. Nevertheless, in recent years, young triathletes at around 20 years of age have been achieving excellent results at the highest-level races, and hence the importance of updating the understand of this aspect.

### 3.2. Elite Triathlete Anthropometric Profile

It seems that there are no anthropometric characteristics directly related to success. Most studies that analyzed the anthropometric characteristics of elite triathletes focused mainly on height, weight and adipose level, either expressed in fat percentage or sum of folds (see Table 2). Although these values are usually present in most studies, it is difficult to find lean mass data. Authors that analyzed this factor in elite triathletes indicated significant relationships of some anthropometric parameters, such as low-fat percentage and long body segments (arms for swimming and legs for cycling and running) [10,11,12,13]. In particular, large dimensions of hands (length: 19.7 ± 0.7 male and 18.2 ± 1.0 female) and feet (shoe size 43.3 ± 2.8 male and 38.3 ± 1.6 female) [14] can especially influence swimming.

The average in women’s height over the years has remained around 167 cm; however, a decrease in weight can be observed from the beginning of the century (~60 kg) to present (~55 kg). This decrease is partly due to the decrease in fat percentage around mean values of 19.27% ± 1.94% [13,15,16,17]. In males, the average height has always been close to 180 cm, while weight has remained around 70 kg. Recently, the fat percentage has reduced below 10%, at around 8.77% ± 1.62% [8,13,15,16,17,18,19,20,21,22,23,24,25,26] (see Table 2).

**Table 2:** Mean ± SD values for the anthropometric profile of elite triathletes.

Authors	N	Age (Years)	Weight (kg)	Height (cm)	Σ Folds (mm)	Fat (%)
González-Parra et al. [17]	2 ♀	23.0 ± 4.2	54.5 ± 3.3	168.5 ± 9.2	-	16.6 ± 0.7
Schabort et al. [16]	5 ♀	25 ± 7	59.3 ± 5.8	167 ± 4.2	-	19.5 ± 2.4
Canda et al. [13]	26 ♀	25.6 ± 4.3	53.8 ± 3.8	163.2 ± 5.4	67.7 ± 17.6 ^(8)^	19.8 ± 3.1
Millet and Bentley [15]	9 ♀	27.9 ± 5.0	60.3 ± 6.6	167.2 ± 5.4	-	21.2 ± 2.9
Ackland et al. [11]	19 ♀	29.0 ± 3.0	59.3 ± 4.7	168.3 ± 4.4	62.8 ± 13.4 ^(8)^	-
Laurenson et al. [27]	10 ♀	27.1 ± 3.5	56.4 ± 6.1	167.0 ± 6.8	25.9 ± 9.4 ^(4)^	-
Werneck et al. [8]	56 ♀	27.7 ± 4.1	54.2 ± 4.5	167 ± 6	-	-
Olaya and Cejuela [24]	4 ♂	22.5 ± 1.9	71.4 ± 4.2	184 ± 41	34.4 ± 1.8 ^(6)^	6.5 ± 0.5
Koury et al. [23]	10 ♂	29 ± 10	69 ± 4	174 ± 5	-	7 ± 2
Zapico et al. [26]	9 ♂	26 ± 2	67.8 ± 2.1	177 ± 20	42.8 ± 3.9 ^(6)^	7.3 ± 0.4
Gonzalez-Haro et al. [21]	6 ♂	25.3 ± 4.2	69.9 ± 4.6	175.2 ± 4.5	38.9 ± 5.7 ^(6)^	7.6 ± 0.6
González-Parra et al. [17]	4 ♂	23.3 ± 2.9	66.7 ± 6.5	167.8 ± 4.4	-	7.8 ± 0.5
Díaz et al. [20]	5 ♂	24.8 ± 5.6	71.9 ± 6.8	172 ± 3	-	8.3 ±0.4
Díaz et al. [19]	6 ♂	24.8 ± 5.6	71.9 ± 6.8	180.2 ± 8.6	-	8.3 ± 0.4
	6 ♂	24 ± 5.6	71.2 ± 8.7	180.0 ± 8.8	-	8.5 ± 0.6
Schabort et al. [16]	5 ♂	23 ± 4	72.1 ± 4.7	181 ± 1.6	-	9.7 ± 2.4
Canda et al. [13]	65 ♂	26.0 ± 4.3	68.5 ± 5.0	178.0 ± 5.2	48.4 ± 9.4 ^(8)^	9.9 ± 2.2
Chollet et al. [18]	6 ♂	24.7 ± 1.3	69.3 ± 1.9	177.5 ± 2.0	-	10.1 ± 0.8
Millet and Bentley [15]	9 ♂	24.8 ± 2.6	70.2 ± 5.2	177.9 ± 4.8	-	10.4 ± 2.1
Hoffmann et al. [22]	11 ♂	23.4 ± 2.8	74.5 ± 4.3	187.0 ± 2.90	-	10.7 ± 1.4
Park et al. [25]	8 ♂	23.5 ± 3.6	66.0 ± 5.1	174.4 ± 4.9	-	11.8 ± 0.5
Ackland et al. [11]	19 ♂	26.3 ± 4.4	72.6 ± 6.0	180.1 ± 5.9	48.3 ± 10.2 ^(8)^	-
Hue [28]	8 ♂	24.8 ± 2.1	71.4 ± 7.3	180.5 ± 9.3	22.3 ± 0.5 ^(4)^	-
Werneck et al. [8]	55 ♂	28.3 ± 4.2	67.6 ± 5.3	180 ± 6	-	-

♀ (females); ♂ (males); Σ folds ^(number of folds)^.

The triathletes’ anthropometric profile does not seem to be defined by height or weight (i.e., great female triathletes under 160 cm in height or male Olympic champions weighing in at 80 kg). Therefore, despite the diversity of profiles, this aspect should not be exclusive, and rather should be considered, since long segments or low-fat levels can facilitate performance in any of the disciplines.

### 3.3. Elite Triathlete Physiological Profile

Among the main factors that influence triathlon performance, physiological parameters are the commonly most explored. Many authors directly relate successful performance to different physiological parameters, most of them giving great importance to maximum oxygen consumption (VO_2_max) relative to weight and the percentage of VO_2_max that triathletes can maintain (ventilatory threshold intensity) [29,30]. These are considered as the primary indicators of triathlon performance success, hence their large-scale study.

Other parameters such as movement efficiency, VO_2_ kinetics or anaerobic capacity have been scarcely studied, and therefore it is difficult to state their influence on elite female and male triathlon performance.

#### 3.3.1. Maximum Oxygen Consumption (VO_2_max) and Ventilatory Threshold (TV2)

The VO_2_max is one of the most commonly studied parameters in triathletes, both absolute and relative to weight; however, greater importance is given to the latter [10,29,31]. The cardiac adaptations produced by endurance training such as increases in stroke volume, due to the enlargement in size and mass of the left ventricle, causes an increase in VO_2_max [30]. Most authors that have reported VO_2_max values for cycling and running disciplines obtained them in laboratory settings using cycle ergometers and treadmills, respectively. These ergometers used for obtaining VO_2_ data are widely used in both triathlon training and research.

Female elite triathletes’ VO_2_max mean values are around 67.3 ± 23.79 mL·kg^−1^·min^−1^, both in treadmill and cycle ergometer tests [15,16,17,27,32]. However, very few females’ data have been reported, so these values should be considered with caution. Regarding males, values usually exceed 70 mL·kg^−1^·min^−1^, with mean values of 72.90 ± 3.96 mL·kg^−1^·min^−1^ [15,16,17,19,20,21,22,24,26,28,32,33,34,35,36,37] (see Table 3).

As with VO_2_max, ventilatory threshold 2 (VT2) is another of the most influential parameters in triathlon performance. Triathletes complete more than half of the race at an intensity above VT2 [36]. Hence, VO_2_ values at VT2 intensity close to VO_2_max will allow triathletes to maintain high-intensity exercise for a longer time, which will increase the possibility of obtaining a better performance [10,29,30,31]. A compilation of triathletes’ VO_2_ values at VT2 intensity is summarized in Table 4. It is worth noting the scarce presence of data on elite female triathletes, with only one study reporting values over 80% of VO_2_max [15]. In males, the mean value is 84.41% ± 2.72% [15,20,21,24,26,31], although great variation can be observed, with values ranging between 81% and 87% of VO_2_max (see Table 4).

#### 3.3.2. Cardiac Parameters

The physiological adaptations induced by training in the cardiovascular system produce an improvement in triathlon performance [30]. The relationship between heart rate (HR) and VO_2_ is widely used to control training intensity. The behavior of this parameter has been studied by numerous authors, both in competition and in laboratory. From our knowledge, the reserve HR value is the best indicator for triathlon performance, but few studies report this parameter, instead focusing on HRmax (see Table 5).

In a World Cup race, Bernard et al. [32] reported that the mean beats per minute (bpm) in the cycling segment was 165 ± 5, which represents 91% ± 4% of the HRmax of the triathletes analyzed. Whyte et al. [38] defined the characteristics of physiological myocardial hypertrophy presented by potential Olympians and/or members of British squads regarding interventricular wall thickness during diastole (male: 10.4 ± 1.5 mm; female: 9.2 ± 0.9 mm); left ventricular internal diameter during diastole (male: 54.9 ± 3.1 mm; female: 50.2 ± 2.1 mm); ventricular posterior free wall during diastole (male: 9.8 ± 1.5 mm; female: 9.3 ± 0.9 mm); and left ventricular mass (male: 274 ± 64 g; female 207 ± 34 g). These authors considered the abovementioned values as high and associated with the concurrent effect of resistance and endurance necessary for good performance in triathlons.

To our knowledge, only case studies have analyzed HR variability in elite short-distance triathletes. Plews et al. [39] verified the changes produced during a 77-day training period in two elite triathletes (1 female and 1 male). One was diagnosed with overtraining during the study period, presenting a higher coefficient of variation for rMSSD (calculated as the root mean of the standard deviations of the differences between successive cycles, measured in ms), as well as lower values than those of the healthy triathlete and even its own values at the beginning of the season. These data show that the increase in rMSSD is an indicator of good physical shape compared to baseline values out of season, and its variations may be indicative of poor adaptation or overtraining.

#### 3.3.3. Respiratory Parameters

The possibility of predicting performance through peak values of pulmonary ventilation has been highlighted [29].

Boussana et al. [40] studied elite triathletes’ respiratory muscles’ resistance, observing how these high-level triathletes are able to breathe against a submaximal inspiratory load for longer time (*p* < 0.05), both before and after exercising compared to lower-level triathletes. These authors pointed out the greater adaptive capacity of elites’ respiratory muscles’ resistance compared to lower-level triathletes. These adaptations can be produced by a higher training load or more years of practice [40].

Maximum lung ventilation (VEmax) has mainly been studied in treadmill tests. Laurenson et al. [27] obtained in elite females a mean VEmax of 123 ± 19.7 L·min^−1^, showing no significant differences with lower-level triathletes. In elite males, Hue et al. [35] reported mean values of 140.1 ± 17.8 L·min^−1^ on a treadmill and 135.5 ± 22.0 L·min^−1^ on a cycle ergometer, with no significant differences between both. However, these authors did find higher values in lower-level triathletes (144.0 ± 18.4 L·min^−1^ and 143.4 ± 22.3 L·min^−1^, respectively).

Another parameter analyzed in elite triathletes was the gas exchange index, which refers to the relationship between exhaled CO_2_ and inhaled O_2_. Hue et al. [35] studied this parameter in males, both in cycle ergometer and treadmill tests, obtaining mean values of 1.14 ± 0.04 and 1.11 ± 0.4, respectively, without significant differences between both types of tests. These authors also analyzed the tidal volume of elite triathletes during maximum effort, also showing no significant differences between values obtained in cycle ergometer (3.02 ± 0.59 L) and treadmill (2.77 ± 0.34 L) tests in elite males. However, there were differences (*p* = 0.01) when the sample included non-elite triathletes. Respiratory frequency was also addressed by Hue et al. [35], obtaining in elite males, at maximum effort, mean values of 45.3 ± 4.L breaths·min^−1^ and 51.1 ± 6.4 breaths·min^−1^ in cycle ergometer and treadmill tests, respectively, showing significant differences (*p* = 0.02) between tests.

#### 3.3.4. Blood Parameters

Blood parameters were measured under competition conditions and/or simulation. Park et al. [25] analyzed the cardiac damage biomarkers after performing an Olympic distance triathlon. These authors found significant decreases in lactate dehydrogenase and troponin cTnT in elite triathletes, and yet there were no variations in creatine kinase, creatine isoenzyme CK-MB, myoglobin and HCT.

Blood lactate concentration ([La]) has been studied in elite male triathletes during simulated competition conditions, reporting mean [La] values of 6.8 ± 2.1 mmol·L^−1^ after the swimming segment (1500 m in a pool) and 5.2 ± 1.5 mmol·L^−1^ after the cycling segment (60 min at maximum speed) [21]. After 20 min of running at 74.4% ± 4.9% of VO_2_max and preceded by 30 min of cycling at 71.7% ± 9.8% of VO_2_max, a [La] of 2.6 ± 1.1 mmol·L^−1^ was reported [28]. However, there is a relationship between running performance and the ideal energy parameter for clearing lactate. It appears that for elite males, −50% of VT1 was found to be the most effective intensity for lactate elimination while running [33].

#### 3.3.5. Hormonal Parameters

Two of the most studied hormones in the field of sports are cortisol and testosterone. The salivary concentrations of these hormones appear to be possible predictors of triathlon performance, since the increase in early morning salivary cortisol concentration, but not the testosterone/cortisol ratio, could be used to predict performance in athletes during a professional triathlon competition [41].

Triathletes’ main physiological adaptations are typical of an endurance sport. Even so, being a long-duration sport, it can be observed that in competitions and/or simulations, HR and [La] are quite high, despite its 2 h duration. This may indicate that the interaction and the differentiated muscular involvement between segments allows well-trained triathletes to maintain very high ranges of effort for long periods of time. These adaptions are achieved with years of training, but during triathletes’ growth, it is essential to consider how these adaptations evolve during youth development phases or training processes.

### 3.4. Biomechanical and Neuromuscular Factors

Mechanical movement is of great importance in any sport. In triathlons, technique or power development seem to have a great impact on performance. Therefore, the technical mastery of each discipline will determine the overall performance in the triathlon [42]. Notwithstanding, the technical ability of cycling and running is influenced by the exertion of the previous discipline, hence the technical differences observed when these sports are performed in isolation [2].

Likewise, movement efficiency is highly related to performance in any of the three disciplines. The ability to exercise at low VO_2_max percentage to achieve a submaximal workload is essential in successful triathlon performance [10,43]. The cardiovascular, metabolic, and neuromuscular adaptations produced by training cause high-level triathletes to increase their movement economy [10,30,31].

#### 3.4.1. Swimming

The swimming segment in triathlon competitions is carried out in open environments, hence the great differences from performing in a pool. Therefore, it is necessary that triathletes adapt their swimming technique to the different water conditions and the possibility for swim drafting [18].

Another aspect that has a great impact on technique is the equipment used in the competition. One of the most analyzed elements is the wetsuit [44]. Its use is determined by the water and environmental temperature, as regulated by the *World Triathlon*. Swimming parameters without drafting or the wetsuit are reported in Table 6. These values vary according to distance and conditions.

Stroke rate (SR) is the number of complete arm cycles performed in a minute (cycles·min^−1^). No studies were found that measured this parameter in elite females without facilitated conditions (see Table 6). In elite males, the maximum value found was 40.0 ± 1.1 cycles·min^−1^ in a 400 m test [18]; however, the mean was 37.9 ± 1.9 cyles·min^−1^ [18,21,44].

Stroke length (SL) is how far the triathlete advances in each stroke (m·cycle^−1^). Different authors studied this parameter, specifically to analyze differences between swimming tactics. In Table 6, mean values of 2.27 ± 0.2 m·cycle^−1^ over 800 m [44] and 1.18 ± 0.1 m·cycle^−1^ over 400 m [16] can be observed for elite males; however, the mean length is 1.91 ± 0.4 m·cycle^−1^ [16,18,21,28,44]. In elite females, this parameter decreases to mean values of 1.02 ± 0.04 m per cycle over 400 m [16]. The differences between studies may be due to how the authors calculated the SL. Hue et al. [44] and Chollet et al. [18], who obtained similar values, divided the average speed of each length by the number of strokes. However, Schabort et al. [16] counted the number of strokes per length and divided by the meters of each length.

Stroke index (SI) is calculated by multiplying speed by SL. Triathletes reported an average index of 2.55 ± 0.59 m^2^·s^−1^ [16,18,28,44]. However, the highest value reported in males was 3.09 ± 0.4 m^2^·s^−1^ over 800 m [44], while the lowest was 1.7 ± 0.3 m^2^·s^−1^ over 800 m [16], which may be influenced by SL calculation, as previously pointed out. In elite females, only [16] reported this value (see Table 6).

Hydrostatic lift (HL) is a method of measuring buoyancy measured in Newtons (N). The HL is what allows a body to float when submerged. It is measured with the subject facing down in the fetal position with a lead mass placed (0.1 kg to 1 kg) between the scapula. The final load required to keep a balanced position under the water is considered the HL [45]. This is an easy method with a high reality (r = 0.98) [46]. Elite males’ and females’ values are 2.13 ± 0.5 N [28] and 2.1 ± 0.7 N [45], respectively (see Table 6).

Several authors focused on the effect of swim drafting on the feet. Millet et al. [45] found significant differences (*p* < 0.05) in passive drag, and therefore greater benefit for triathletes when swimming with their hands closer to the feet of the triathlete in front. In this line, Chollet et al. [18] revealed that drafting improves performance by reducing the global energy cost of swimming by 10%, showing significantly lower values (*p* < 0.05) of [La] (9.6 mmol·L^−1^ vs. 10.8 mmol·L^−1^), SL (2.13 m·cycle^−1^ vs. 2.03 m·cycle^−1^) and SI (2.95 m^2^·s^−1^ vs. 2.74 m^2^·s^−1^) without influencing SR.

Regarding the use of a wetsuit, swimming time performance over 400 m was significantly lower with neoprene (19 s), while higher VO_2_ and [La] values were obtained [46]. Therefore, these authors highlighted that to take advantage of the wetsuit, it is important to be used to it, since swimmers do not follow the same trend. Hue et al. [44] did not found significant differences in leg movement at maximum speed over any distance (50, 100 and 800 m), although they found a significant increase (*p* < 0.01) in SI and SL at shorter distances and a shorter impulse phase (*p* < 0.01) and lower coordination index for the 800 m distance.

In brief, it seems that the use of a wetsuit is beneficial for performance. The changes in coordination and the great benefits when accustomed to its use confirm that a specific skill is required to get the most out of the wetsuit.

Triathletes and swimmers have similar gross efficiency, stroke frequency (SF) and watts per stroke; however, SL (1.23 ± 0.21 m swimmers vs. 0.92 ± 0.23 m triathletes) and speed (1.17 ± 0.8 m·s^−1^ vs. 0.95 ± 0.11 m·s^−1^) is higher in swimmers due their propulsion efficiency, because they are able to use more power to overcome drag [47]. In addition, Millet et al. [48] also reported that swimmers are more efficient in the propulsive phase and have significant higher SL than triathletes. These authors also reported that when triathletes reach high swimming speeds, unlike swimmers, they increase the length of the recovery phase. Therefore, the improvement of these aspects can be of great interest for elite triathletes to maximize swim performance, of course considering the characteristics of triathlon swimming.

#### 3.4.2. Cycling

The parameters most often addressed in the analysis of triathlon performance during the cycling segment are power behavior and pedaling cadence.

Millet et al. [31] related race performance to high values of aerobic power peak achieved in the cycle ergometer test. Elite triathletes’ peak power output (PPO) in cycling has been analyzed by various authors (see Table 7). In this type of cycle ergometer test it is also very common to report the power achieved at VT2, both absolute and relative to weight (see Table 8). Nevertheless, to our knowledge, the maximum anaerobic peak has only been analyzed by Bernard et al. [32], reporting values in a maximum sprint of 6 s on a cycle ergometer of 942.8 ± 119.2 W and 14.2 ± 2.0 W·kg^−1^ in males and 676.7 ± 124.6 W and 12.3 ± 1.8 W·kg^−1^ in females. These authors used these data to draw up the power–speed relationship and identified ManP (maximal anaerobic power) as the peak in this relationship. 

In the cycling segment of a male and female World Cup race, a mean total power of 230 ± 53 W (3.6 ± 0.5 W·kg^−1^, which represented 60% ± 8% of the maximum aerobic power of the athletes) [32] and 252 ± 33 W (3.9 ± 0.5 W·kg^−1^) for males was reported [49]. Bernard et al. [32] pointed out that most of the segment (51% ± 9%) was performed at an intensity lower than VT1, 17% ± 6% was between VT1 and VT2, 15% ± 3% between VT2 and PPO, and 6% at an intensity greater than PPO. Etxebarria et al. [49] reported a power coefficient of variation during the race of 71% ± 13% with peaks of more than 600 W and approximately 18% of the time it was performed above the PPO. These later authors suggested through a logarithmic equation that power could be maintained constantly throughout the cycling segment at 291 ± 29 W, which is 40 ± 13 W greater than the real value. A decrease in intensity is therefore observed as the race progresses, progressively increasing the time in the zone below VT1 and decreasing in the other three zones. Thus, it is important to highlight the presence of short explosive moments in which PPO is exceeded. This indicates the need for the ability in the cycling segment to sprint to obtain a better position in the peloton or to not lose contact with it.

Regarding pedaling cadence, significant differences were observed between males (95 ± 4 rpm) and females (88 ± 4 rpm) participating in a World Cup [37], reporting an increase in cadence in males at the end of the segment [50]. In this line, Etxebarria et al. [49] reported an oscillation in males’ average values between 77 and 106 rpm in 12 World Series and World Cup races.

In summary, the cycling segment in elite triathlons is characterized by a variable power, with high-intensity short-duration peaks.

#### 3.4.3. Running

Running performance has been identified as the most decisive discipline in the overall triathlon performance [2]. Therefore, the analysis of running biomechanics and how it is affected by the cycling and swimming segments plays a fundamental role in triathlon performance. Several authors have addressed this issue by performing laboratory tests [51,52,53]. However, it is not very common to find these types of studies in elite competitions.

The most commonly analyzed parameters of triathlon running technique are the stride pattern (length and frequency) and body position. Cala et al. [54] were the first authors to focus on running kinematics at high-level competition (a World Cup), observing significant differences in males’ speed and stride length between the first and the last lap. However, females only showed significant differences in stride length.

Comparing the biomechanical differences, a female World Cup winner showed a greater stride length, lower frequency, greater distance between the vertical projection of the hip and the heel at the strike, together with a larger angle in the extension of the knee of the supporting leg with respect to the toes than the rest of participants [55]. These characteristics can help to define an optimal running pattern, although it would be important to carry out similar studies with a large sample to help to verify whether it is a common pattern. Le Meur et al. [56] found, during a Grand WTS Final, a simultaneous decrease in running speed, vertical angulation, and leg angulation throughout the laps, except for the finishing straight.

Running stride length can explain variation in speed to a greater extent than cadence [56], and it is significantly related to running and overall triathlon performance [50], although it can be modified by stiffness adaptions induced by fatigue [57]. Therefore, the improvement in running performance cannot be explained only by physiological changes, but also by anthropometric and biomechanical parameters [24].

Regarding lower limbs’ muscle activity measured with EMG during a treadmill test, no differences were found between elite triathletes and runners, finding differences only with less trained athletes in the percentage of individual variance (RMSD%) [58]. In any case, following Bonacci et al. [59], there are differences in leg muscle recruitment between multidisciplinary training and a single disciple. However, it is unknown whether these specific neuromuscular adaptations are beneficial or detrimental to sport-specific performance.

In summary, it appears that biomechanical variations produced in the final running segment are influenced by running speed, with a high stride length being the factor most related to performance. To our knowledge, there are no studies that have analyzed, with the same triathletes, running biomechanics without previous cycling. This would help one to know if the running pattern is influenced by previous cycling.

### 3.5. Elite Triathletes Tactical Strategy

Despite each race having specific characteristics [60], it is very important to know the trends in competition strategy to measure the effort throughout the race. Therefore, the multidisciplinary nature of the triathlon and the interaction of the three disciplines must be considered to establish a global strategy.

#### 3.5.1. Relative Importance of Each Discipline

Many authors have addressed the importance of each discipline to the overall result in triathlon. There are mainly two fields of thought: on the one hand, that the most decisive segment in the final triathlon result is the cycling segment [12,61], and on the other hand, that the running is the most influential discipline in the overall triathlon performance [2,7,62,63]. However, Gadelha et al. [64] reported that during the “drafting period”, swimming was the discipline with the highest influence on overall race time for females (only when ranked ≥4th place), although over an Olympic distance, no differences between males and females were found by Sousa et al. [65].

Regarding the influence of the final position in the cycling segment and the final triathlon result, high correlations have been found in elite females (r = 0.904) [12], mainly due to the greater number of breaks or cycling packs [61,62,63]. However, it has been concluded that cycling performance is less important than the other two disciplines [2,66], it being more important for women than for men [63], even though it is the discipline in which the highest percentage of time is invested (53.07%) [67]. However, females spend more time than males (both in this and the rest of the segments) [68].

Nevertheless, considering studies that analyzed recent high-level races, it seems that the weight of cycling performance has increased [61,65]. Olaya et al. [61] concluded that cycling performance is the most influential in the overall triathlon result. However, Sousa et al. [65] considered that the combination of cycling and running has the most influence on the overall triathlon result for both females and males in standard and sprint distance. This suggests that, as has been happening for a decade in females, the cycling segment has gained greater importance in the final result. During the “drafting period”, this segment seemed to be a pathway for males between the swimming and the running; however, now we can see that cycling packs are less numerous, it being crucial to be fighting for the higher positions.

There seem to be more authors confirming that running is more decisive in the final triathlon result. High correlations were obtained in elite males between each segment position and the overall result (r = 0.810–0.94) [12,62,64,66,67,69,70,71].

However, in females, these correlations are slightly lower (r = 0.71–0.85) [62,64,69]. Therefore, the difference in overall performance in a top-level triathlon is mostly given by performance in the running segment [2,7,63,72].

However, small losses of seconds in the swimming segment and transitions can have a significant impact on the final result [62]. Hence, despite the short duration of the transitions (0.74% in T1 and 0.47% in T2), their importance in the final result is quite high [67,70].

In brief, running performance is the discipline that most determines the final result in high-level competitions, especially between 1998 and 2000 [73], becoming more important over the years [74]. This should be considered for competitions and training, obviously without neglecting the rest of the disciplines. Swimming and cycling (and even transitions) are not that decisive in the overall result of the current triathlon, but a bad segment can leave a triathlete without any hope in the race.

#### 3.5.2. Competition Strategy

Understanding the importance of each discipline helps athletes to set objectives in the split times that will influence the final triathlon result and to make tactical decisions to optimize performance [2]. Thus, triathletes with lower swimming performance need higher cycling performance in the first kilometers of the segment, requiring them to use cycling tactics to maximize the effort that will affect the subsequent running, and therefore the triathlon performance [75].

Focusing on the swimming segment, no differences have been observed in the overall average speed between triathletes who finish in the top 50% or the final 50% [66]. These authors also found a correlation between finishing the race in the top 50% and being one of the fastest triathletes in the initial meters (222 m and 496 m) (*p* < 0.01, r = −0.88 and r = −0.97 males and females, respectively) [69]. The speed of the first 350 m of swimming was faster (*p* < 0.05) than the rest of the segment in all triathletes, since the position after those first meters strongly determines the final position in the swim, for both females and males (r = 0.97 and 0.99, *p* < 0.01, respectively) [37]. However, the swimming final position has a low correlation with the final running position (r = 0.47 and 0.36, *p* < 0,01 females and males, respectively) [37]. Therefore, although the swimming does not have a great influence on the overall triathlon position, both a bad start and a low speed in the first meters of this first segment can mean losing any hope of being in the higher positions at the end of the swim.

In the cycling segment, a positive strategy has been observed, among both females and males, with a significant decrease in pace by lap more pronounced among males [32,37]. This progressive reduction in speed is related to preparing the strategy of the next segment [32].

Regarding the running segment, the first part is run faster than the rest of the segment [37,69]. However, when triathletes have direct opponents in the final meters of the race, the running speed is increased [76], adopting a positive strategy [37,56,71]. Therefore, this shows that an aggressive racing strategy is usually adopted by elite triathletes, with a high speed at the beginning of the segment that is gradually reduced and influenced by high levels of motivation and the presence of rivals.

More research is needed to determine whether these conclusions are influenced by the race dynamics or are extensible to other conditions (i.e., type of circuit, participants, weather conditions, etc.) [37]. Triathlon is a very young sport with continuous variations in competition rules, where increasingly shorter distances, such as team relay or sprint distance, have greater weight at international level. This makes triathletes’ competition strategy very changeable, since the relative importance of each discipline varies between Olympic cycles. Therefore, it is essential that elite triathletes need to have good performance in all disciplines to be able to face any international-level race with confidence.

Despite being long-duration races, during all disciplines, there are periods of very high intensity, especially at the beginning of the segment. For this reason, it is very important that triathletes who seek to maximize their performance need to be able to reach and perform at very high speeds, being capable of maintaining these paces without compromising the rest of the race, whatever the competition strategy. Losing contact with the leading group in any of the segments makes it practically impossible to reach a good final position.

### 3.6. Elite Triathletes Psychosocial Profile

Research on psychological and social factors in triathlon is very scarce, and studies that relate these factors directly to performance are even rarer. The most influential positive factors for triathletes, both males and females, and coaches are dedication/commitment, perseverance, and work willingness [77]. These authors reported that the main negative factors are the injuries, together with a lack of personal confidence and competitive pressure. Notably, elite triathletes’ psychological factors seem to be taken care of by professionals, since 64% of females and 60% of males reported seeing a psychotherapist [14].

Elite triathletes revealed that they give importance to their mental health. However, much remains to be explored to define the psychological profile that makes triathletes reach the highest level. There are many triathletes who, despite having great physical qualities, are not capable of reaching the elite due to mental issues.

### 3.7. Interactions between Disciplines

The peculiarity of the triathlon is its chaining of three disciplines without a rest between them. The aim that various authors seek is to understand the effect that each discipline has on subsequent performance, although most of these authors did not include elite triathletes in their studies. To our knowledge, all studies with elites focus on T2 (transition from cycling to running), pointing out the requirements of specific biomechanical, physiological, and sensory adaptations [1]. These authors highlighted the importance of the ability to optimally chain these two disciplines (cycling and running) to improve running efficiency, which, together with the great influence of the final segment in the overall triathlon performance, makes it crucial.

#### 3.7.1. Cycling Effect on Running Motor Coordination

No variations were found in neuromuscular control that could affect performance when running after cycling in high-level triathletes, although these variations were found in lower-level triathletes (see Table 9) [53].

#### 3.7.2. Cycling Effect on Running Cardiorespiratory Response

In the previous research, no significant differences were found in any of the cardiorespiratory parameters measured during running with and without previous cycling in elite triathletes [19,34] (see Table 9). However, they did find significant differences in lower-lever triathletes [34], and even in young triathletes [19]. This indicates that elite triathletes have greater cardiorespiratory adaptations than lower-level triathletes. In addition, these adaptions have not yet occurred in young triathletes, despite their high level at their age, which reflects the need to accumulate years of training to achieve these adaptations. However, significant differences have been found in [La] values, both in elite and lower-level triathletes, which probably increase due to the [La] during the cycling segment [51].

Pulmonary responses in elite triathletes during T2 show that cycling produces a decrease in the diffusing capacity of the lungs for carbon monoxide (D_LCO_) in both elite and lower-level triathletes, although in elites, this effect does not persist during running. Hence, it can be concluded that higher-level triathletes have specific responses to carry out T2 with greater guarantees.

#### 3.7.3. Cycling Effect on Running Energy Cost

The running energy cost (EC) is frequently used to determine differences between isolated running and transition [15,28,31]. To calculate energy cost variation (ΔEC) produced by previous cycling, the following formula (1) is usually used [15]:

ΔEC = (EC_run2_ − EC_run1_)/EC_run1_ (1)

According to Cala et al. [54], previous cycling does not affect running efficiency because at the end of the run, this efficiency is even lower, probably due to fatigue and the decrease in speed and stride length. Therefore, these authors state that it cannot be attributed on the cycling segment. To deepen this statement, studies that compare running patterns with and without previous cycling would be necessary to fully understand the cycling segment’s effects on the running segment.

However, cycling and running’s gross efficiencies (calculated as the ratio between the mechanical work rate and metabolic rate) are positively correlated (r = 0.66; *p* = 0.038; R^2^ 0.44) [78]—that is, triathletes with a high cycling efficiency are also efficient runners.

In summary, elite triathletes hardly show variations in running biomechanics and cardiorespiratory response between previous cycling and in isolation. However, in some cases, variations in motor coordination were observed. Therefore, the main difference between elite and less experienced triathletes is their ability to adapt to running with high levels of fatigue caused by previous cycling in a way that affects the run segment as little as possible.

### 3.8. Performance Prediction in Competition

Different authors have identified different parameters that can predict triathlon performance in competitions [7,16,22,28,41].

On one hand, it has been pointed out that parameters from laboratory and field tests such as cycling and running [La] (during a cycling test at a stable state 4 W·kg^−1^ and a 15 km·h^−1^ running test; r = 0.92 and 0.89, respectively), cycling PPOmax (r = 0.86) and maximum running peak speed (r = 0.85) significantly helped (*p* < 0.01) to predict the total time of the running segment [16]. These authors established Formula (2) with high significance (r = 0.90, *p* < 0.01):

Running time (s) = −129 (running peak velocity [km·h^−1^] + 122 ([La] at 4 W·kg^−1^) + 9456 (2)

Similarly, to predict Olympic distance triathlon results with drafting using specific laboratory tests, Hue [28] also established Formula (3), mainly through a 30 min cycling test and a 20 min running test (C-R):

Triathlon time (s) = −1018 (achieved distance in R in B-R [m]) + 39.1 ([La] at final R in B-R) + 12,518(3)

In addition, cortisol upon waking (r = 0.79, *p* < 0.05) and 30 min post competition (r = 0.76, *p* < 0.05) also appears to help predict competition performance [41], as well as anthropometric (weight, BMI, fat% and lean body mass) and physiological (VO_2_max, running pace at 3 mmol·L^−1^ [La], maximum running pace) parameters [22]. However, results evolution in high-level competition can also predict results in future competitions [7].

It seems that there are a series of parameters that, when measured in the short term, can help to predict elite female and male triathletes’ performance in competitions. However, it is important to know whether these parameters are related in the long term, which could be very useful for triathlon talent detection programs.

### 3.9. Talent Identification Programs

“One of the most dominant success indicators of a single nation in triathlon is the number of athletes on the highest level of sports performance” [79]. These authors highlighted that a nation’s success primarily depends on their long-term strategy development system, which implies investing in the youngest female and male age groups and supporting the coaches, who play essential roles in identifying, motivating and attracting talent.

To our knowledge, there are few authors in the scientific literature that address the talent identification processes in triathlon. Bottoni et al. [80] identified the main factors to consider in triathlon talent identification programs, pointing out that they should not be based only on physical parameters, but also on variables of different kinds. Other authors, such as Cuba-Dorado et al. [81,82], based their research on the explanatory capacity of the Spanish talent identification battery test, highlighting the low capacity to predict performance. However, Phillips and Newland [83] focused on institutional aspects, analyzing the agents involved in the Australian and USA programs, while Ortigosa-Marquez et al. [84] and Ferriz-Valero et al. [85] analyzed the influence of relative age on the detection and development processes of young triathletes.

Much remains to be explored to achieve the fairest possible identification and development programs for young female and male triathlon talents. It is essential that those who oversee their implementation (i.e., coaches, technicians, federations, researchers, etc.) must consider the factors that determine triathlon performance (i.e., physiological, anthropometric, psychosocial, and tactical factors) and the competition demands. Currently, the triathlon is subjected to constant regulatory changes, where major competitions tend to adopt shorter distances to seek greater spectacle. This requires constant updates, forcing triathletes to adapt to these new demands, which will possibly lead to a growing trend of explosive female and male triathletes with an earlier age of specialization. This should undoubtedly be considered for talent development programs.

This review exclusively focuses on elite female and male triathlete characteristics to serve as a basis for designing innate talent identification tests that are tailored to the current reality of the triathlon. However, for the successful development of these young talents, it is also essential to delve into other aspects, such as the optimal age to work out each of these characteristics (i.e., how and when young female and male triathletes should start training for that purpose), what factors can limit their performance, etc. Therefore, further research should also consider these issues to provide greater knowledge in the field and better tools for coaches.

Finally, this study aims to be a starting point to profile the special characteristics that triathletes must have to reach a high performance. It is essential that talent identification programs locate the main factors that determine high performance. However, when designing global programs, these must be longitudinal and not only regarding to talent identification, but also physical and mental development. Hence, future research lines should focus more on these concerns rather than only the special characteristics that can lead young triathletes to reach a high level of performance.

## 4. Conclusions

The main findings of this narrative review indicate that there are physiological, biomechanical, neuromuscular, tactical parameters, etc., that could be related to successful triathlon performance in competitions.

***Competition Age.*** It seems that the optimal age for female and male triathlon performance is about 30. However, continuous updates in this regard are essential due to the results achieved in recent years by young triathletes.

***Anthropometric profile.*** The anthropometric profile of elite triathletes does not seem to be defined by height or weight, mainly due to the diversity of profiles. Therefore, this aspect should not be exclusive in talent identification programs, but should be considered since long segments or low-fat levels can facilitate performance in any of the disciplines.

***Physiological profile*.** Triathletes’ main physiological adaptations are typical of an endurance sport. One of the most studied parameters is the VO_2_max. Female elite triathletes’ relative VO_2_max values are around 67.3 ± 23.79 mL·kg^−1^·min^−1^, while males usually exceed 70 mL·kg^−1^·min^−1^. The VT2 has also been widely studied in elite triathletes, reporting values over 80% of VO_2_max for females and 84.41% ± 2.72% of VO_2_max for males. However, being a long-duration sport, it can be observed that in competitions and/or simulations, HR and [La] are quite high. This may indicate that the interaction and differentiated muscular involvement between the swimming, cycling and running segments allows very well-trained triathletes to maintain very high ranges of effort for long periods of time. These characteristic adaptions are mainly achieved with training, but when it comes to young triathletes’ development it is essential to consider how these adaptations will evolve over the years.

***Biomechanical and neuromuscular factors.*** The fact that the swimming takes places in open environments means that there are differentiating aspects to consider regarding to swim in a pool. The cycling segment in elite triathlon is characterized by a variable power with high intensity short-duration peaks. The running segment is influenced by the previous disciplines; however, highly experienced triathletes can adapt themselves to this last segment, despite the high levels of fatigue accumulated, so that it affects them as little as possible.

***Tactical Strategy***. The increasing importance of shorter distances (i.e., team relay or sprint distance) for the *World Triathlon* makes triathletes’ competition strategy very changeable, which affects the relative importance of each discipline. Therefore, elite triathletes need to have good performance in all disciplines to be able to face any international level race with confidence.

***Interaction between disciplines.*** The main difference between elite and less experienced triathletes is their ability to adapt to running with high levels of fatigue caused by previous cycling in a way that affects the running segment as little as possible.

***Talent Identification programs.*** It is essential that when implementing talent identification programs, these factors that determine triathlon performance (i.e., physiological, anthropometric, psychosocial, and tactical factors) and the competition demands are considered. However, constant updating is needed due the continuous evolution of regulatory changes and the needs of triathletes to adapt to these new competition demands.

## Figures and Tables

**Table 1:** Official distances in triathlon.

	Swim	Cycle	Run
Team Relay	250 m to 300 m	5 km to 8 km	1.5 km to 2 km
Super Sprint Distance	250 m to 500 m	6.5 km to 13 km	1.7 km to 3.5 km
Sprint Distance	Up to 750 m	Up to 20 km	Up to 5 km
Standard Distance *	1500 m	40 km	10 km
Middle Distance	1900 m to 3000 m	80 km to 90 km	20 km to 21 km
Long Distance	1000 m a 4000 m	100 km a 200 km	10 km a 42.2 km

* Olympic Distance.

**Table 3:** Mean ± SD values of VO_2_max reported for elite triathletes.

Authors	Laboratory Test	N	Age (Years)	Weight (kg)	VO_2_max (mL·kg^−1^·min^−1^)	VO_2_max (L·min^−1^)
Bernard et al. [32]	Cycle ergometer	3 ♀	26.9 ± 4.7 *	55 ± 2.6	67.3 ± 0.7	-
Schabort et al. [16]	Cycle ergometer	5 ♀	25 ± 7	59.3 ± 5.8	61.3 ± 4.6	3.6 ± 0.4
Millet and Bentley [15]	Cycle ergometer	9 ♀	27.9 ± 5.0	60.3 ± 6.6	61.0 ± 5.0	3.7 ± 0.4
Le Meur et al. [37]	Cycle ergometer	6 ♀	27 ± 4	57 ± 5	60.9 ± 7.0	-
Díaz et al. [19]	Cycle ergometer	6 ♂	24 ± 5.6	71.2 ± 8.7	77.8 ± 3.6	-
	Cycle ergometer	6 ♂	24.8 ± 5.6	71.9 ± 6.8	77.4 ± 4.6	-
Díaz et al. [20]	Cycle ergometer	5 ♂	24.8 ± 5.6	71.9 ± 6.8	77.6 ± 5.1	4.9 ± 0.2
Hue et al. [35]	Cycle ergometer	6 ♂	21.8 ± 2.4	69.9 ± 7.3	75.9 ± 5.2	5.3 ± 0.4
Hue et al. [34]	Cycle ergometer	5 ♂	25.4 ± 0.8	72.2 ± 3.4	75.7 ± 2.3	-
Millet and Bentley [15]	Cycle ergometer	9 ♂	24.8 ± 2.6	70.2 ± 5.2	74.3 ± 4.4	5.2 ± 0.3
Zapico et al. [26]	Cycle ergometer	9 ♂	26 ± 2	67.8 ± 2.1	72.9 ± 2.0	4.9 ± 0.2
Le Meur et al. [37]	Cycle ergometer	6 ♂	30 ± 6	67 ± 5	71.7 ± 5.4	-
Hue [28]	Cycle ergometer	8 ♂	24.7 ± 2.1	71.4 ± 7.3	70.5 ± 6.5	-
Schabort et al. [16]	Cycle ergometer	5 ♂	23 ± 4	72.1 ± 4.7	69.9 ± 4.5	5.0 ± 0.4
Bernard et al. [32]	Cycle ergometer	5 ♂	26.9 ± 4.7 *	67 ± 5	69.8 ± 5.3	
Gonzalez-Haro et al. [21]	Cycle ergometer	6 ♂	25.3 ± 4.2	69.9 ± 4.6	64.7 ± 5.7	4.6 ± 0.3
Hue et al. [36]	Cycle ergometer	5 ♂	25.7 ± 1	71.6 ± 3.3	64.4 ± 1.2	-
González-Parra et al. [17]	Treadmill	2 ♀	23.0 ± 4.2	54.5 ± 3.3	74.0 ± 0.1	-
Laurenson et al. [27]	Treadmill	10 ♀	27.1 ± 3.5	56.4 ± 6.1	65.6 ± 6.0	-
Schabort et al. [16]	Treadmill	5 ♀	25 ± 7	59.3 ± 5.8	63.2 ± 3.6	3.7 ± 0.3
Hue et al. [35]	Treadmill	6 ♂	21.8 ± 2.4	69.9 ± 7.3	78.5 ± 3.6	5.5 ± 0.3
Hue et al. [34]	Treadmill	5 ♂	25.4 ± 0.8	72.2 ± 3.4	76.3 ± 3.2	-
González-Parra et al. [17]	Treadmill	4 ♂	23.3 ± 2.9	66.7 ± 6.5	76.0 ± 6.9	-
Schabort et al. [16]	Treadmill	5 ♂	23 ± 4	72.1 ± 4.7	74.7 ± 5.3	5.3 ± 0.5
Olaya-Cuartero and Cejuela [24]	Treadmill	4 ♂	22.5 ± 1.9	71.4 ± 4.2	72.8 ± 2.2	-
Hoffmann et al. [22]	Treadmill	11 ♂	23.4 ± 2.8	74.5 ± 4.3	72.0 ± 4.3	5.5 ± 0.3
Hue [28]	Treadmill	8 ♂	24.7 ± 2.1	71.4 ± 7.3	71.8 ± 7.6	-
Baldari et al. [33]	Treadmill	8 ♂	21 ± 1	73 ± 4	69.7 ± 4.7	-
Hue et al. [36]	Treadmill	5 ♂	25.7 ± 1	71.6 ± 3.3	69.5 ± 1	-

VO_2_max (maximum oxygen consumption); ♀ (females); ♂ (males); * (mean age from overall sample: female + male).

**Table 4:** Mean ± SD values of VO_2_ at VT2 of elite triathletes.

Authors	Laboratory Test	N	Age (Years)	Weight (kg)	VT2 (mL·kg^−1^·min^−1^)	VT2 (%VO_2_max)
Millet and Bentley [15]	Cycle ergometer	9 ♀	27.9 ± 5.0	60.3 ± 6.6	-	80.5 ± 7.9
Zapico et al. [26]	Cycle ergometer	9 ♂	26 ± 2	67.8 ± 2.1	-	86.2 ± 1.6
Millet and Bentley [15]	Cycle ergometer	9 ♂	24.8 ± 2.6	70.2 ± 5.2	-	83.9 ± 4.5
Gonzalez-Haro et al. [21]	Cycle ergometer	6 ♂	25.3 ± 4.2	69.9 ± 4.6	-	83 ± 5
Díaz et al. [20]	Cycle ergometer	5 ♂	24.8 ± 5.6	71.9 ± 6.8	-	81.0 ± 4.4
Olaya and Cejuela [24]	Treadmill	4 ♂	22.5 ± 1.9	71.4 ± 4.2	64 ± 2.94	87.94 ± 1.59
Baldari et al. [33]	Treadmill	8 ♂	21 ± 1	73 ± 4	52.9 ± 4	-

VT2 (ventilatory threshold 2); ♀ (females); ♂ (males).

**Table 5:** Mean ± SD values of HRmax of elite triathletes.

Authors	Laboratory Test	N	Age (Years)	Weight (kg)	HRmax (bpm)
Bernard et al. [32]	Cycle ergometer	3 ♀	26.9 ± 4.7 *	55 ± 2.6	185.7 ± 13.1
Millet y Bentley [15]	Cycle ergometer	9 ♀	27.9 ± 5.0	60.3 ± 6.6	184.3 ± 7.1
Millet y Bentley [15]	Cycle ergometer	9 ♂	24.8 ± 2.6	70.2 ± 5.2	187.6 ± 8.9
Díaz et al. [19]	Cycle ergometer	6 ♂	24 ± 5.6	71.2 ± 8.7	186 ± 3
	Cycle ergometer	6 ♂	24.8 ± 5.6	71.9 ± 6.8	184 ± 4
Zapico et al. [26]	Cycle ergometer	9 ♂	26 ± 2	67.8 ± 2.1	183 ± 5
Bernard et al. [32]	Cycle ergometer	5 ♂	26.9 ± 4.7 *	67 ± 5	180.8 ± 5.4
Hue et al. [36]	Cycle ergometer	5 ♂	25.7 ± 1	71.6 ± 3.3	177 ± 3
González-Haro et al. [21]	Cycle ergometer	6 ♂	25.3 ± 4.2	69.9 ± 4.6	176 ± 14
Hue et al. [35]	Cycle ergometer	6 ♂	21.8 ± 2.4	69.9 ± 7.3	174 ± 3
Díaz et al. [20]	Cycle ergometer	5 ♂	24.8 ± 5.6	71.9 ± 6.8	172 ± 3
Laurenson et al. [27]	Treadmill	10 ♀	27.1 ± 3.5	56.4 ± 6.1	186.6 ± 4.9
Olaya and Cejuela [24]	Treadmill	4 ♂	22.5 ± 1.9	71.4 ± 4.2	191 ± 9.3
Hue et al. [35]	Treadmill	6 ♂	21.8 ± 2.4	69.9 ± 7.3	184 ± 5
Hue et al. [36]	Treadmill	5 ♂	25.7 ± 1	71.6 ± 3.3	182 ± 5

HRmax (maximum heart rate); bpm (beats per minute); ♀ (females); ♂ (males); * (mean age from overall sample: female + male).

**Table 6:** Mean ± SD of swimming biomechanical parameters in elite triathletes.

Authors	N	Distance (m)	Time (s)	Vel (m·s^−1^)	SR (cyles·min^−1^)	SL (m·cyles^−1^)	SI (m^2^·s^−1^)	SV (m·s^−1^)	HL (N)
Schabort et al. [16]	5 ♀	400	326 ± 28	1.23 ± 0.10 *	-	1.02 ± 0.04	1.3 ± 0.1	-	-
Hue [28]	8 ♂	400	288 ± 12	1.39 ± 0.06 *	-	1.97 ± 0.2	2.7 ± 0.2	-	2.13 ± 0.5
Chollet et al. [18]	6 ♂	400	283.7 ± 4.1 *	1.39 ± 0.02	40.0 ± 1.1	2.03 ± 0.06	2.74 ± 0.02	-	1.9
Schabort et al. [16]	5 ♂	400	279 ± 19	1.43 ± 0.09 *	-	1.18 ± 0.10	1.7 ± 0.3	-	-
Hue et al. [44]	12 ♂	800	590 ± 15	1.36 ± 0.03 *	36.4 ± 4.2	2.27 ± 0.2	3.09 ± 0.4	1.36 ± 0.03	-
Gonzalez-Haro et al. [21]	6 ♂	1500	1102.9 ± 59.9 *	1.29 ± 0.07	37.2 ± 3.2	2.09 ± 0.18	-	-	-

Vel (velocity); SR (stroke rate); SL (stroke length); SI (stroke index); SV (stroke velocity; HL (hydrostatic lift); ♀ (females); ♂ (males); * (calculated from authors data).

**Table 7:** Mean ± SD of peak power output (PPO) in cycle ergometer tests of elite triathletes.

Authors	Protocol	N	Age (Years)	Weight (kg)	PPO_rel_ (W·kg^−1^)	PPO (W)
Bernard et al. [32]	W_i_ = 100 W for 6’; t_e_ = 2’; W_e_ = 25 W	3 ♀	26.9 ± 4.7 *	55 ± 2.6	5.4 ± 0.3	296.3 ± 29.7
Le Meur et al. [37]		6 ♀	27 ± 4	57 ± 5	5.2 ± 0.2	293 ± 19
Millet and Bentley [15]	W_i_ = 70 W for 3’; t_e_ until 280 W = 3’, then 2’; W_e_ up to 280 W = 70 W then 35 W	9 ♀	27.9 ± 5.0	60.3 ± 6.6	4.8 ± 0.4	292.8 ± 20.9
Bernard et al. [32]	W_i_ = 100 W for 6’; t_e_ = 2’; W_e_ = 30 W	5 ♂	26.9 ± 4.7 *	67 ± 5	6.3 ± 0.6	418.0 ± 26.8
Le Meur et al. [37]		6 ♂	30 ± 6	67 ± 5	6.2 ± 0.2	418 ± 22
González-Haro et al. [21]	W_i_ = 100 W for 10’; t_e_ = 4’; W_e_ = 30 W	6 ♂	25.3 ± 4.2	69.9 ± 4.6	4.9 ± 0.3	345 ± 14
Díaz et al. [19]	W_i_ = 75 W for 5’; t_e_ = 60 s; W_e_ = 25 W	6 ♂	24 ± 5.6	71.2 ± 8.7	5.7 ± 1.2	-
		6 ♂	24.8 ± 5.6	71.9 ± 6.8	5.9 ± 0.8	-
Millet and Bentley [15]	W_i_ = 70 W for 3’; t_e_ until 280 W = 3’, then 2’; W_e_ up to 280 W = 70 W then 35 W	9 ♂	24.8 ± 2.6	70.2 ± 5.2	5.5 ± 0.6	384.7 ± 50.2
Hue et al. [36]	W_i_ = 30 W for 3’; t_e_ = 1’; W_e_ = 30 W	5 ♂	25.7 ± 1	71.6 ± 3.3	-	389 ± 24
Zapico [26]	W_i_ = 0 W for 1’; t_e_ = 1’; W_e_ = 25 W	9 ♂	26 ± 2	67.8 ± 2.1	5.9 ± 1.5	402 ± 23.0

PPO (Peak power output); W (watts); W_i_ (initial watts); t_e_ (time per step); W_e_ (increment at each step); ♀ (females); ♂ (males); * (mean age from overall sample: female + male).

**Table 8:** Mean ± SD power values at VT2 of elite triathletes.

Authors	N	Age (Years)	Weight (kg)	Power VT2 (W·kg^−1^)	Power VT2 (W)
Bernard et al. [32]	3 ♀	26.9 ± 4.7 *	55 ± 2.6	-	241.7 ± 14.4
Le Meur et al. [37]	6 ♀	27 ± 4	57 ± 5	-	232 ± 24
	6 ♂	30 ± 6	67 ± 5	-	349 ± 22
Bernard et al. [32]	5 ♂	26.9 ± 4.7 *	67 ± 5	-	336.0 ± 23.0
Zapico [26]	9 ♂	26 ± 2	67.8 ± 2.1	-	336 ± 13.5
González-Haro et al. [21]	6 ♂	25.3 ± 4.2	69.9 ± 4.6	-	298 ± 40
Díaz et al. [19]	6 ♂	24.8 ± 5.6	71.9 ± 6.8	3.6 ± 1.0	-
	6 ♂	24 ± 5.6	71.2 ± 8.7	3.4 ± 0.8	-
Hue [28]	8 ♂	24.7 ± 2.1	71.4 ± 7.3	3.3 ± 0.5	-

VT2 (Ventilatory Threshold 2); ♀ (females); ♂ (males); * (mean age from overall sample: female + male).

**Table 9:** Effect of cycling on the running performance of elite triathletes.

Authors	N	Elite Age (Years)	Protocol	Effect
Bonacci et al. [53]	7 elite (4 ♂ + 3 ♀)	24.9 ± 3.7 *	5’ R at submaximal speed (16 km/h ♀ and 18 km/h ♂) 1. Ri; 2. 20’ B at low intensity + R; 3. 50’ B at high intensity + R.	No significant differences in muscular recruitment and joint angles between protocols.
Díaz et al. [19]	15 ♂ (6 elite + 9 youth)	24.8 ± 5.6 24.0 ± 5.6	30’ B at VT + 3 km R at max speed, other session 3 km Ri at max speed. Carried out over 2 consecutive years with the same triathletes.	Differences only in elites’ speed (higher in Ri) and HR (lower in the 1st year).
Hue et al. [34]	13 ♂ (5 elite + 8 comp)	25.4 ± 0.8	30’ B + 1’ T + 20’ R and on a different day 20’ Ri at same speed at VT.	No significant differences between Ri and B-R in VO_2_, V_E_, V_E_/VO_2_, V_E_/VCO_2_, R, f_R_, V_T_, and HR.
Hue [28]	8 elite ♂	24.7 ± 2.1	30’ B max speed + 20’ speed based on Olympic triathlon performance and 20’ R at the same speed.	At the same speed, there are hardly any variations in VO_2_ y EC. Higher V_E_ in Ri with respect to T.
Hue el al. [52]	14 ♂ (6 elite + 8 comp)	23.1 ± 1.2	30’ B + 1’ T + 20’ R and on a different day 30’ B at the same speed at VT. Lung function was evaluated before and after each test.	Increases in V_E_, V_E_/VO_2_, V_E_/VCO_2_, f_R_ and HR in elites’ R with respect to B. At the beginning of R, there is a decrease in D_LCO_, but it does not persist over time.
Millet et al. [31]	15 elite ♂ (9 SD + 6 LD)	24.8 ± 2.6	Continuously (1’ T): 7’ Ri short triathlon intensity + Test MAP B +10’ B at 80%MAP + 7’ R at Ri speed.	No significant differences in elites’ SD in EC before and after the maximum B test.
Millet et al. [15]	18 elite (9♂ + 9 ♀) 13 juniors (7♂ + 6 ♀)	27.9 ± 5.0 ♀ 24.8 ± 2.6 ♂	Same protocol as Millet et al. [31]	Lower running EC in T than in Ri in elite ♀ The opposite occurs in the rest.
Millet et al. [51]	8 elite (1 ♂ + 7 ♀) 18 no-elite (14 ♂+ 4 ♀)	29 ± 3 ♀ 31 ♂	Same protocol as Millet et al. [31]	No significant changes between Ri and R in HR, V_E_, VO_2_ and running EC. There is an increase in [La] in R.

R (run); B (bike); Ri (isolated R without previous B); B-R (R with previous B); T (transition); SD: standard distance; LD: long distance; VT (Ventilatory threshold); HR (heart rate); [La] (lactate concentration); V_E_ (lung ventilation); VO_2_ (oxygen consumption); VCO_2_ (carbon dioxide production); D_LCO_ (diffusing capacity of the lungs for carbon monoxide); EC (energy cost); ♀ (females); ♂ (males); * (mean age from overall sample: male + female).

## Data Availability

The data presented in this study are available on request from the corresponding author.

## References

[1] Millet G.P., Vleck V.E. (2000). Physiological and biomechanical adaptations to the cycle to run transition in Olympic triathlon: Review and practical recommendations for training. Br. J. Sports Med..

[2] Ofoghi B., Zeleznikow J., Macmahon C., Rehula J., Dwyer D.B. (2016). Performance analysis and prediction in triathlon. J. Sports Sci..

[3] Bentley D.J., Bishop D. (2008). Science and medicine of triathlon. J. Sci. Med. Sport.

[4] Vaeyens R., Lenoir M., Williams A.M., Philippaerts R.M. (2008). Talent identification and development programmes in sport—Current models and future directions. Sports Med..

[5] Johnston K., Wattie N., Schorer J., Baker J. (2018). Talent Identification in Sport: A Systematic Review. Sports Med..

[6] Villaroel C., Mora R., González-Parra G.C. (2011). Elite triathlete performance related to age. J. Hum. Sport Exerc..

[7] Malcata R.M., Hopkins W.G., Pearson S.N. (2014). Tracking career performance of successful triathletes. Med. Sci. Sports Exerc..

[8] Werneck F.Z., Lima J.R.P., Coelho E.F., Matta M., Figueiredo A.J.B. (2014). Relative age effect on olympic triathlon athletes. Rev. Bras. Med. Esporte.

[9] Knechtle R., Rüst C.A., Rosemann T., Knechtle B. (2014). The best triathletes are older in longer race distances—A comparison between Olympic, Half-Ironman and Ironman distance triathlon. SpringerPlus.

[10] Sleivert G.G., Rowlands D.S. (1996). Physical and physiological factors associated with success in the triathlon. Sports Med..

[11] Ackland T.R., Blanksby B.A., Landers G., Smith D. (1998). Anthropometric profiles of elite triathletes. J. Sci. Med. Sport.

[12] Landers G.J., Blanksby B.A., Ackland T.R., Smith D. (2000). Morphology and performance of world championship triathletes. Ann. Hum. Biol..

[13] Canda A.S., Castiblanco L.A., Toro A.N., Amestoy J.A., Higueras S. (2014). Morphological characteristics of the triathlete according to sex, category and competitive level. Apunt. Med. L’esport.

[14] Brunkhorst L., Kielstein H. (2013). Comparison of anthropometric characteristics between professional triathletes and cyclists. Biol. Sport.

[15] Millet G.P., Bentley D.J. (2004). The physiological responses to running after cycling in elite junior and senior triathletes. Int. J. Sports Med..

[16] Schabort E.J., Killian S.C., Gibson A.S., Hawley J.A., Noakes T.D. (2000). Prediction of triathlon race time from laboratory testing in national triathletes. Med. Sci. Sports Exerc..

[17] González-Parra G., Mora R., Hoeger B. (2013). Maximal oxygen consumption in national elite triathletes that train in high altitude. J. Hum. Sport Exerc..

[18] Chollet D., Hue O., Auclair F., Millet G., Chatard J.C. (2000). The effects of drafting on stroking variations during swimming in elite male triathletes. Eur. J. Appl. Physiol..

[19] Diaz V., Peinado A.B., Vleck V.E., Alvarez-Sanchez M., Benito P.J., Alves F.B., Calderon F.J., Zapico A.G. (2012). Longitudinal changes in response to a cycle-run field test of young male national “talent identification” and senior elite triathlon squads. J. Strength Cond. Res..

[20] Díaz V., Martínez E.D., Peinado A.B., Benito P.J., Calderón F.J., Sampedro J. (2010). Biological control of training during the precompetitive period in elite triathletes: A pilot study. Arch. Med. Deporte.

[21] Gonzalez-Haro C., Gonzalez-de-Suso J.M., Padulles J.M., Drobnic F., Escanero J.F. (2005). Physiological adaptation during short distance triathlon swimming and cycling sectors simulation. Physiol. Behav..

[22] Hoffmann M., Moeller T., Seidel I., Stein T. (2017). Predicting elite triathlon performance: A comparison of multiple regressions and artificial neural networks. Int. J. Comput. Sci. Sport.

[23] Koury J.C., De Oliveira Jr A.V., Portella E.S., De Oliveira C.F., Lopes G.C., Donangelo C.M. (2004). Zinc and copper biochemical indices of antioxidant status in elite athletes of different modalities. Int. J. Sport Nutr. Exerc. Metab..

[24] Olaya-Cuartero J., Cejuela R. (2021). Influence of Biomechanical Parameters on Performance in Elite Triathletes along 29 Weeks of Training. Appl. Sci..

[25] Park C.H., Kim K.B., Han J., Ji J.G., Kwak Y.S. (2014). Cardiac damage biomarkers following a triathlon in elite and non-elite triathletes. Korean J. Physiol. Pharmacol..

[26] Zapico A.G., Benito P.J., Diaz V., Ruiz J.R., Calderon F.J. (2014). Heart rate profile in highly trained triathletes. Rev. Int. Med. Cienc. Act. Fis. Deporte.

[27] Laurenson N.M., Fulcher K.Y., Korkia P. (1993). Physiological characteristics of elite and club level female triathletes during running. Int. J. Sports Med..

[28] Hue O. (2003). Prediction of drafted-triathlon race time from submaximal laboratory testing in elite triathletes. Can. J. Appl. Physiol..

[29] Kovářová L., Kovář K. (2012). Verification of the model of predisposition in triathlon—Structural model of confirmative factor analysis. Acta Univ. Palacki. Olomuc. Gymnica.

[30] O’ Toole M.L., Douglas P.S. (1995). Applied physiology of triathlon. Sports Med..

[31] Millet G.P., Dreano P., Bentley D.J. (2003). Physiological characteristics of elite short- and long-distance triathletes. Eur. J. Appl. Physiol..

[32] Bernard T., Hausswirth C., Le Meur Y., Bignet F., Dorel S., Brisswalter J. (2009). Distribution of Power Output during the Cycling Stage of a Triathlon World Cup. Med. Sci. Sports Exerc..

[33] Baldari C., Videira M., Madeira F., Sergio J., Guidetti L. (2005). Blood lactate removal during recovery at various intensities below the individual anaerobic threshold in triathletes. J. Sports Med. Phys. Fit..

[34] Hue O., Le Gallais D., Boussana A., Chollet D., Prefaut C. (2000). Performance level and cardiopulmonary responses during a cycle-run trial. Int. J. Sports Med..

[35] Hue O., Le Gallais D., Chollet D., Prefaut C. (2000). Ventilatory threshold and maximal oxygen uptake in present triathletes. Can. J. Appl. Physiol..

[36] Hue O., Galy O., Le Gallais D. (2006). Exercise intensity during repeated days of racing in professional triathletes. Appl. Physiol. Nutr. Metab..

[37] Le Meur Y., Hausswirth C., Dorel S., Bignet F., Brisswalter J., Bernard T. (2009). Influence of gender on pacing adopted by elite triathletes during a competition. Eur. J. Appl. Physiol..

[38] Whyte G.P., George K., Sharma S., Firoozi S., Stephens N., Senior R., McKenna W.J. (2004). The upper limit of physiological cardiac hypertrophy in elite male and female athletes: The British experience. Eur. J. Appl. Physiol..

[39] Plews D.J., Laursen P.B., Kilding A.E., Buchheit M. (2012). Heart rate variability in elite triathletes, is variation in variability the key to effective training? A case comparison. Eur. J. Appl. Physiol..

[40] Boussana A., Hue O., Matecki S., Galy O., Ramonatxo M., Varray A., Le Gallais D. (2002). The effect of cycling followed by running on respiratory muscle performance in elite and competition triathletes. Eur. J. Appl. Physiol..

[41] Balthazar C.H., Garcia M.C., Spadari-Bratfisch R.C. (2012). Salivary concentrations of cortisol and testosterone and prediction of performance in a professional triathlon competition. Stress- Int. J. Biol. Stress.

[42] Bentley D.J., Millet G.P., Vleck V.E., McNaughton L.R. (2002). Specific aspects of contemporary triathlon—Implications for physiological analysis and performance. Sports Med..

[43] Hausswirth C., Lehenaff D. (2001). Physiological demands of running during long distance runs and triathlons. Sports Med..

[44] Hue O., Benavente H., Chollet D. (2003). The effect of wet suit use by triathletes: An analysis of the different phases of arm movement. J. Sports Sci..

[45] Millet G., Chollet D., Chatard J.C. (2000). Effects of drafting behind a two- or a six-beat kick swimmer in elite female triathletes. Eur. J. Appl. Physiol..

[46] Chatard J.C., Senegas X., Selles M., Dreanot P., Geyssant A. (1995). Wet suit effect—A comparison between competitive swimmers and triathletes. Med. Sci. Sports Exerc..

[47] Toussaint H.M. (1990). Differences in propelling efficiency between competitive and triathlon swimmers. Med. Sci. Sports Exerc..

[48] Millet G.P., Chollet D., Chalies S., Chatard J.C. (2002). Coordination in front crawl in elite triathletes and elite swimmers. Int. J. Sports Med..

[49] Etxebarria N., D’Auria S., Anson J.M., Pyne D.B., Ferguson R.A. (2014). Variability in power output during cycling in international olympic-distance triathlon. Int. J. Sports Physiol. Perform..

[50] Landers G.J., Blanksby B.A., Rackland T. (2011). Cadence, Stride Rate and Stride Length during Triathlon Competition. Int. J. Exerc. Sci..

[51] Millet G.P., Millet G.Y., Hofmann M.D., Candau R.B. (2000). Alterations in running economy and mechanics after maximal cycling in triathletes: Influence of performance level. Int. J. Sports Med..

[52] Hue O., Galy O., Le Gallais D., Prefaut C. (2001). Pulmonary responses during the cycle-run succession in elite and competitive triathletes. Can. J. Appl. Physiol..

[53] Bonacci J., Saunders P.U., Alexander M., Blanch P., Vicenzino B. (2011). Neuromuscular control and running economy is preserved in elite international triathletes after cycling. Sports Biomech..

[54] Cala A., Veiga S., Garcia A., Navarro E. (2009). Previous cycling does not affect running efficiency during a triathlon world cup competition. J. Sports Med. Phys. Fit..

[55] Cala A., Cejuela R., Veiga S., Garcia A., Navarro E., Perez Turpin J.A. Biomechanical Analysis of The Running Part at Competition Triathlon World Cup. Differences Between Men and Women. Proceedings of the 1st Joint International Pre-Olympic Conference of Sports Science and Sports Engineering.

[56] Le Meur Y., Thierry B., Rabita G., Dorel S., Honnorat G., Brisswalter J., Hausswirth C. (2013). Spring-mass behaviour during the run of an international triathlon competition. Int. J. Sports Med..

[57] Rabita G., Slawinski J., Girard O., Bignet F., Hausswirth C. (2011). Spring-Mass Behavior during Exhaustive Run at Constant Velocity in Elite Triathletes. Med. Sci. Sports Exerc..

[58] Chapman A.R., Vicenzino B., Blanch P., Hodges P.W. (2008). Is running less skilled in triathletes than runners matched for running training history?. Med. Sci. Sports Exerc..

[59] Bonacci J., Chapman A., Blanch P., Vicenzino B. (2009). Neuromuscular Adaptations to Training, Injury and Passive Interventions Implications for Running Economy. Sports Med..

[60] Bentley D.J., Cox G.R., Green D., Laursen P.B. (2008). Maximising performance in triathlon: Applied physiological and nutritional aspects of elite and non-elite competitions. J. Sci. Med. Sport.

[61] Olaya J., Fernández-Sáez J., Østerlie O., Ferriz-Valero A. (2021). Contribution of segments to overall result in elite triathletes: Sprint distance. Int. J. Environ. Res. Public Health.

[62] Cejuela R., Cortell-Tormo J.M., Chinchilla-Mira J.J., Pérez-Turpin J.A., Villa J.G. (2012). Gender differences in elite Olympic distance triathlon performances. J. Hum. Sport Exerc..

[63] Piacentini M.F., Bianchini L.A., Minganti C., Sias M., Di Castro A., Vleck V. (2019). Is the Bike Segment of Modern Olympic Triathlon More a Transition towards Running in Males than It Is in Females?. Sports.

[64] Gadelha A.B., Sousa C.V., Sales M.M., dos Santos Rosa T., Flothmann M., Barbosa L.P., da Silva Aguiar S., Olher R.R., Villiger E., Nikolaidis P.T. (2020). Cut-Off Values in the Prediction of Success in Olympic Distance Triathlon. Int. J. Environ. Res. Public Health.

[65] Sousa C.V., Aguiar S., Olher R.R., Cunha R., Nikolaidis P.T., Villiger E., Rosemann T., Knechtle B. (2021). What Is the Best Discipline to Predict Overall Triathlon Performance? An Analysis of Sprint, Olympic, Ironman® 70.3, and Ironman® 140.6. Front. Physiol..

[66] Vleck V.E., Burgi A., Bentley D.J. (2006). The consequences of swim, cycle, and run performance on overall result in elite olympic distance triathlon. Int. J. Sports Med..

[67] Cejuela R., Cala A., Perez-Turpin J.A., Villa J.G., Cortell J.M., Chinchilla J.J. (2013). Temporal Activity in Particular Segments and Transitions in The Olympic Triathlon. J. Hum. Kinet..

[68] Lepers R., Knechtle B., Stapley P.J. (2013). Trends in triathlon performance: Effects of sex and age. Sports Med..

[69] Vleck V.E., Bentley D.J., Millet G.P., Bürgi A. (2008). Pacing during an elite Olympic distance triathlon: Comparison between male and female competitors. J. Sci. Med. Sport.

[70] Cejuela R., Perez Turpin J.A., Cortell J.M., Villa Vicente J.G. An Analysis of Transition Time in the World Championship of Triathlon—Hamburg 2007: Determination of the Lost Time T2. Proceedings of the 1st Joint International Pre-Olympic Conference of Sports Science and Sports Engineering.

[71] Etxebarria N., Wright J., Jeacocke H., Mesquida C., Pyne D.B. (2021). Running your best triathlon race. Int. J. Sports Physiol. Perform..

[72] Figueiredo P., Marques E.A., Lepers R. (2016). Changes in Contributions of Swimming, Cycling, and Running Performances on Overall Triathlon Performance Over a 26-Year Period. J. Strength Cond. Res..

[73] Gonzalez-Haro C., De Suso Janáriz J.M.G. (2002). Gestión de la competición durante el triatlón de distancia olímpica. Análisis de los resultados de las temporadas 1998, 1999 y 2000. Apunt. Educ. Fis. Y Deportes.

[74] Rüst C.A., Lepers R., Stiefel M., Rosemann T., Knechtle B. (2013). Performance in Olympic triathlon: Changes in performance of elite female and male triathletes in the ITU World Triathlon Series from 2009 to 2012. Springerplus.

[75] Bentley D.J., Vleck V.E. (2004). Pacing strategy and performance in elite World Cup triathlon: A preliminary study. Med. Sci. Sports Exerc..

[76] Le Meur Y., Bernard T., Dorel S., Abbiss C.R., Honnorat G., Brisswalter J., Hausswirth C. (2011). Relationships between triathlon performance and pacing strategy during the run in an international competition. Int. J. Sports Physiol. Perform..

[77] Ruiz-Tendero G., Salinero Martin J.J. (2012). Psycho-social factors determining success in high-performance triathlon: Compared perception in the coach-athlete pair. Percept. Mot. Ski..

[78] Carlsson M., Wahrenberg V., Carlsson M.S., Andersson R., Carlsson T. (2020). Gross and delta efficiencies during uphill running and cycling among elite triathletes. Eur. J. Appl. Physiol..

[79] Kasović M., Škrinjarić B., Štefan L. (2020). Macro and meso indicators of success pertaining to European countries in elite triathlon. Sport Sci..

[80] Bottoni A., Gianfelici A., Tamburri R., Faina M. (2011). Talent selection criteria for olympic distance triathlon. J. Hum. Sport Exerc..

[81] Cuba-Dorado A., García-García O., Hernández-Mendo A. (2015). Explanatory capacity triathletes performance through talent detection of Spanish federation. Cuad. Psicol. Deporte.

[82] Cuba-Dorado A., Garcia-Garcia O., Morales-Sánchez V., Hernández-Mendo A. (2020). The Explanatory Capacity of Talent Identification Tests for Performance in Triathlon Competitions: A Longitudinal Analysis. J. Hum. Kinet..

[83] Phillips P., Newland B. (2014). Emergent models of sport development and delivery: The case of triathlon in Australia and the US. Sport Manag. Rev..

[84] Ortigosa-Marquez J.M., Reigal R.E., Serpa S., Hernandez-Mendo A. (2018). Relative age effect on national selection process in triathlon. Rev. Int. Med. Cienc. Act. Fis. Deporte.

[85] Ferriz Valero A., Selles Perez S., Garcia Jaen M., Cejuela Anta R. (2020). Relative age effect for talents’ development in joung triathletes. Retos-Nuevas Tend. Educ. Fis. Deporte Recreacion.

